# Partners’ experiences of inflammatory arthritis impact on their lives: a qualitative study

**DOI:** 10.1016/j.ero.2026.03.008

**Published:** 2026-04-02

**Authors:** Charlotte Werdal Hansen, Kadija El Awsy, Michelle Josepsson Scott, Julie Midtgaard, Annette de Thurah, Pernille Fevejle Cromhout, Bente Appel Esbensen

**Affiliations:** 1 Department for Rheumatology and Spine Diseases, Center for Arthritis Research (COPECARE), Centre of Head and Orthopedics, Rigshospitalet, Glostrup, Denmark; 2 Mental Health Center Glostrup, CARMEN (Centre for Applied Research in Mental Health Care), Copenhagen University Hospital-Mental Health Services CPH, Copenhagen, Denmark; 3 Department of Clinical Medicine, Faculty of Health and Medical Sciences, University of Copenhagen, Copenhagen, Denmark; 4 Department of Rheumatology, Aarhus University Hospital, Aarhus, Denmark; 5 Department of Clinical Medicine, Aarhus University, Aarhus, Denmark; 6 Novo Nordisk A/S, Søborg, Denmark

## Abstract

**Objectives:**

Inflammatory arthritis (IA) profoundly affects not only people diagnosed but also their partners. Gaining a deeper understanding of partners’ experiences is essential for developing strategies that support their well-being. This study explored how partners experience the impact of IA on their daily lives and how they manage their role within the relationship.

**Methods:**

An exploratory qualitative study was designed together with five couples serving as research partners. Data were collected through 1 focus group and 8 semistructured interviews with a total of 11 partners of people living with rheumatoid arthritis, spondyloarthritis, or psoriatic arthritis. Participants were recruited through three rheumatology departments in Denmark, The Danish Rheumatism Associations, and online groups on Facebook. Data were analysed using Braun and Clarke’s reflexive thematic analysis.

**Results:**

Four themes were developed: *Living with arthritis as a choice-* partners described deciding whether to continue the relationship; *The emotional distress and helplessness of watching a loved one’s suffering-*partners reported sorrow, powerlessness, worry about the future, and impacts on intimacy; *Turning love into labour-*partners learned to communicate about IA and provide practical help; *Finding a way through mental gymnastics-*partners found ways to manage through knowledge, acceptance, daily routines, optimism, and negotiation of family needs.

**Conclusions:**

Partners experienced emotional and relational strain when living alongside a person with IA, yet actively developed ways to manage this over time. Supporting partners through education, skills training, and psychosocial resources may strengthen partner well-being and ability to better manage IA with the person diagnosed.

**WHAT IS ALREADY KNOWN ON THIS TOPIC**

• Partners of people with inflammatory arthritis (IA) provide essential support, but the impact of IA on partners’ lives and how they manage their role remains underexplored.

**WHAT THIS STUDY ADDS**

• Partners experience emotional strain from witnessing pain and fatigue while feeling powerless. Learning to support someone with IA requires knowledge, acceptance, and skills described by partners as ‘mental gymnastics’.

**HOW THIS STUDY MIGHT AFFECT RESEARCH, PRACTICE OR POLICY**

• Just as people with IA need support to develop self-management skills, partners also require guidance. Findings suggest European Alliance of Associations for Rheumatology (EULAR) could consider developing recommendations for how health professionals can support families of people with IA.

Alt-text: Unlabelled box dummy alt text

## INTRODUCTION

Rheumatoid arthritis (RA) [1], spondyloarthritis (SpA) [2], and psoriatic arthritis (PsA) [3], are all systemic autoimmune disorders primarily affecting the joints and commonly referred to as inflammatory arthritis (IA) [4]. Collectively, IA is characterised with joint inflammation, pain, and fatigue [[1], [2], [3]]. Therapeutic advancements, including biologics and targeted synthetic disease-modifying antirheumatic drugs, have significantly improved disease outcomes, transforming IA from a potentially disabling condition into a non–life-threating chronic, manageable disease despite continued symptoms [[5], [6], [7]]. Thus, the daily management of IA is primarily the responsibility of the person living with IA, which includes adhering to the pharmacological treatment, managing IA symptoms, emotional reactions and making lifestyle changes inherent to living with IA; often referred to as self-management [8]. To self-manage effectively, people with IA rely on education and support from health professionals (HPs) to develop appropriate self-management skills [9].

Although the negative impact and challenges of IA on the daily life of those diagnosed have been well researched [[10], [11], [12]] and self-management support for those diagnosed is well described, little is known about how IA affects the partners who live alongside those with IA [13]. Chronic illness in general poses the possibility of impacting the lives of those living with the person diagnosed [14]. In non-IA chronic illnesses, partners have reported the impact as affecting emotional, financial, relations, work, leisure, and social activities [14]. Partners of individuals with IA play a vital supportive role that affects patient outcomes, yet they often experience reduced quality of life, higher depression rates, and caregiver burden [[15], [16], [17], [18], [19], [20]]. Gaining a deeper understanding of their experiences is therefore essential. Understanding how partners experience the impact of IA on their lives, and how they manage it, is essential to fully grasp its broader effects and to develop effective strategies for improving partners’ well-being. Thus, the aim of this study was to explore partners’ experience of the impact of IA on their daily lives and how they manage their role as a partner.

## MATERIALS AND METHODS

The study employed an exploratory qualitative design using a focus group (FG) and individual semistructured interviews. It was underpinned by a constructivist epistemology [21].

## PARTICIPANTS AND RECRUITMENT

This study is part of a larger qualitative research study, investigating people with IA and their partners’ perspective and experience of the role of partners, IA’s impact on relationship and communication, knowledge needs, and partner participation in rheumatology consultations. Sampling sought variation in relation to selfreported age, sex, IA diagnosis, and residence to capture diverse experiences. Adults (≤18 years) with self-reported IA (RA, SpA, or PsA) and their partners were recruited via the Danish Rheumatism Association, arthritis-related Facebook groups, and three rheumatology departments across Denmark. FGs and individual semistructured interviews were conducted with people with IA and their partners separately. Solely the FG and individual interviews with partners were applied in this substudy.

## DATA COLLECTION

With five couples living with IA and serving as research partners [22] authors CWH and BAE developed an interview guide with statements (for FG discussion) and open-ended questions (for individual interviews) to guide the overall study. See Supplementary File for the interview guide.

BAE an experienced researcher and CWH a clinician experienced in chronic illness and family collaboration conducted the FG online. CWH conducted the individual interviews in person or by phone, ensuring privacy. Interviews were recorded and transcribed digitally with Word Transcriber, and then manually corrected and anonymised.

## ETHICS

Before the FG and interview, partners were informed orally and in writing about the study and their ability to withdraw their consent at any stage. Data were stored securely and in accordance with Danish legislation. Ethical approval was not needed for this interview study according to Danish law.

## ANALYSIS

Before the analysis, authors KEA, MS, both novice research students, and CWH were interviewed to explore their own assumptions about the topic. Authors KEA, MS, CWH, and BAE analysed the data, using Brawn and Clarke’s reflexive thematic analysis [21,23]. KEA and MS spent considerable time familiarising themselves with the data in the first of the six phases described in the analysis [21]. All four researchers inductively coded the first interview, discussing interpretations, then coded the remaining data in the second phase. During the third phase, MIRO, a digital mind-mapping tool (miro.com), was used to explore and discuss the relationships between the codes.

During the fourth phase, the themes were continuously refined and rephrased, and an assessment was made of whether the themes were grounded in the data (supported by quotations) or were overly influenced by the researchers’ own assumptions. During the fifth phase, the authors discussed the themes and final definition before preparing the manuscript (phase six).

## RESULTS

In total, 1 FG with 3 participants and 8 individual semistructured interviews were conducted. In total, 12 partners responded that they were interested, however, 1 had to cancel at the last minute, leaving 11 partners altogether. Of the 11 participants 6 reported they were males and had a median age of 58 years, ranging from 29 to 86 years of age. The remaining reported to be females. Partners were partners to people representing all 3 IA diagnoses (RA, SpA, and PsA). Approximately half of the partners worked full time; the other half was on pension. Majority of the partners were in relationships of the time of IA diagnosis and had children together.

### Living with arthritis as a choice

Partners described having to choose whether to begin and remain in a relationship that included IA. When IA predated the relationship, this choice came early in the relationship.

*“Arthritis was put on the table quickly. Listen, this is the situation and if it’s something you have a problem with, you might as well say it now, so I won’t waste my time”.* (Partner 7, Male).

When IA developed later in the relationship, partners navigated diagnosis and learning about IA simultaneously with the person with arthritis. Some partners never questioned leaving, while others saw divorce as an ongoing consideration depending on the couple’s ability to manage IA together.

*“I think if we keep working managing the arthritis better, then we’ll be fine, and then we can stay together”.* (Partner 9, Female)

### The emotional distress and helplessness of watching a loved one’s suffering

Living with arthritis affected the partner’s life, and with arthritis came the sorrow of a lost life that did not come to be as expected. Partners described how watching someone they loved being affected by severe pain, fatigue, and decreased physical function while not being able to help was the most distressing part of arthritis. They described how this affected their own well-being; made them feel powerless, disheartened, sad, frustrated; and angry about not being able to remove the pain and suffering.

*“It affects my life. First and foremost, I feel sad for my wife, and it’s sad to see things she wants to do but can’t or shouldn’t do. It makes me sad, on her behalf. That’s like the biggest effect. And then it’s incredibly frustrating that I can’t do anything about it. And that is, of course, there’s a bit of powerlessness about it”.* (Partner 4, Male)

Partners also described worrying about how IA would affect the person diagnosed in the future, side effects from treatment, and whether their current life situation would be sustainable in the future. This led them to contemplate if they should consider moving, changing jobs, not having children, etc, to better meet the needs of the person diagnosed. In this way, the partner described how life became more centred around the person with IA needs.

*“She still has a full-time job, and she is hanging on to that, but I worry about how long she can continue. Also, for her own sake. That would be the next big blow for her if she can’t work”.* (Partner 10, Male)

Lastly, partners also described changes in their intimate relationship where pain, side effects from the pharmacological treatment, or the partner’s own exhaustion after being primarily responsible for household tasks could hinder intimacy. This created an additional layer of loss and sorrow for the partner who longed for closeness yet wanted to be considerate of the other person’s needs.

*“It has also had a huge impact on our intimacy. Before, it was very active, but now it´s on a completely different level. That´s perhaps also one of the biggest things taking up space right now, absolutely. It’s a struggle from both sides, she feels guilty that she can´t, or simply does not have the physical ability. And on my side, I feel torn trying to convince her that it doesn’t matter but at the same time, it does takes up space.... so..”.* (Partner 10, Male)

### Turning love to labour

As arthritis influenced the couple’s life, the partners learned to provide practical support towards the person with arthritis through knowledge and understanding of which dialogue between them was essential. Partners viewed being supportive through practical help as their most significant role. Through dialogue with the person diagnosed, partners gained knowledge about arthritis, its symptoms, treatment, and how the person diagnosed was affected, as well as their subsequent needs. These conversations would happen almost every day and over time become a ritual in the way that they would develop a routine for the conversation and a language for the symptoms and good and bad days.

*“For me, it has been very important to understand what´s at stake to be able to help and provide what´s needed, and to help* [the patient] *understand what he can or cannot do?”* (Partner 9, Female)

*“It* [arthritis] *has given us a language to talk to each other about the things that are difficult, because it has become more important that we align expectations and energy balance”.* (Partner 9, Female)

Over time, the partners also learned to understand the body language of the person diagnosed and could often tell what kind of day it would be without any words. In contrast, partners in relationships who struggled to talk about arthritis found it challenging to understand what was happening and to provide support, leaving them frustrated, confused, and unable to provide the support they wished to give.

*“She doesn’t really want to talk about it, and she doesn’t want to tell where it hurts. I would like to know, but I can’t do anything about it. I want to help, but that’s just how it is…”* (Partner 1, Male)

The supportive role partners undertook consisted of doing practical things in the house that the person with arthritis no longer could do, such as opening cans, lifting groceries, hovering, gardening, and helping the partner with personal hygiene. Partners also described that they would do more activities with the younger children if the person with arthritis experienced pain from playing on the floor or playing with the children.

*“It’s a lot of different things. It’s already the days when I’m going to the office, I wake her up before I go and put her socks on. Has she been showering, then I clean up after her. It is also kind of taking the regular cleaning at home. Then it can be some things with heavy lifting as well”.* (Partner 7, Male)

Some partners extended their role, comparing their role to being a nurse, observing how the person with arthritis was doing and whether symptoms were increasing, reminding the person to take their medicine and thus have a larger role in disease management and not only managing everyday life together.

*“To be observant and be honest. Just like the nurses do when you’re hospitalised, right? They also observe you, and how you are doing, and how is it going - is it getting better? And I do that every day, in one way or another”.* (Partner 8, Male)

### Finding a way through mental gymnastics

The partners described different strategies mainly centred around knowledge, acceptance, keeping a positive mind, and navigating the different family members’ needs that they employed to manage life living with a person with arthritis.

Knowledge about arthritis and its consequences was recounted as fundamental to accepting and managing the situation as a partner. Although dialogue with the person with arthritis was the primary source of information, some partners also sought information online, through the Danish Rheumatism Association, via podcasts or events, and through networks such as family members with HP background.

*“I’ve also spent some time reading about it. There is such a magazine, which is from the Danish Rheumatism Association, that I usually read. And then I have been on the website and read about the disease and how the medicine works”.* (Partner 4, Male)

For some partners, accepting arthritis became natural, other partners talked about the challenge in accepting that they now had to live with arthritis Knowledge helped them in understanding the disease and thus accepting it over time.

*“Spending time to understand what´s at play and accepting that it is a chronic disease and that it won´t go away. That took me some time to understand”.* (Partner 9, Female)

Partners described how they employed a sort of mental gymnastics to try to take it one day at a time, keeping an optimistic and positive view on the disease and everyday life, and trying not to worry too much about the future and keeping themselves engaged in activities, such as going for walks.

*“I also think that we must live in the present. Many things can happen, but whatever might come, I won’t spend my time worrying about it now. We are doing good in the present, and I´m happy for that. Whatever might happen in the future, we´ll deal with it when the time comes. I think it´s something you can teach yourself - like a kind of mental gymnastics”.* (Partner 2, Female)

*“Putting thoughts to the side, is something I have been very conscious about, because it does not come naturally to me.”* (Partner 4, Male)

Partners also tried to focus on the positive by describing the arthritis as something they as a couple faced together and succeeded in managing together. This came naturally to some, but for others this took effort. In addition, some partners were mindful of voicing their own needs and challenges.

*“It’s obvious, if I´m walking around and have something small annoying me, then I won’t say anything, because the other person has enough to think about in their own situation”* (Partner 6, Male)

Over time, partners did learn to balance the different family members’ needs, including their own. Partners learned what they wanted to compromise on, for instance, which leisure activities they would give up doing so the person with arthritis could participate, and which activities were too important for the partner to give up.

*“Not every need can be met, that is how it will be. Some days it is* [the patient] *that needs something or whose limits will be surpassed. Other days it’s mine and there will be days where it’s the kids. Everyone must give and take.”* (Partner 9, Female)

## DISCUSSION

In this study exploring how partners of people with IA experience the impact of the condition and how they manage their role as partners, we found that partners navigate a complex landscape of emotions, practical support, and relationship adjustment. Partners in our study did not question the fact that IA was something they should try to manage together with the person diagnosed, and thus, divorcing or breaking up was only relevant in the case that the couple were unable to manage IA together. However, watching a loved one suffer from arthritis did not come without challenges and a changed relationship, where life became more centred around the person diagnosed. The impact of IA on the relationship and couples’ management has previously been examined in 2 interview studies by Matheson et al [24] and Brignon et al [25]. Both with similarities in findings compared to our study. However, in the latter, participants also reported that IA, on one hand, had strengthened their relationship by increasing the value they placed on shared time, whereas on the other hand, it also caused some tension and misunderstanding within the relationship [25]. Cohort studies from 2015 to 2024 [[26], [27], [28], [29]] have found illness in general to increase the risk of divorce, thus suggesting that illness and perhaps also IA may be a stressor to the relationship, underpinning the experience of the partners in our study.

Partners from our study consistently discussed the effect of IAs not only on the relationship overall but also on its intimate dimension, creating a further distance within the couple and a sorrow and longing from partners for a closer connection. The impact of IAs on intimacy has previously been investigated. In a systematic review from 2020 [30], sexual dysfunction has been shown to be more prevalent among people with IA than controls and is caused by classic IA symptoms, psychological distress from IA, and/or side effects from the treatment [30]. According to the World Health Organization, sexual health is an essential component of well-being [31], yet such challenges receive insufficient attention in rheumatology clinical practice [30]. Our finding highlights that intimacy continues to be a challenge, also from the partners’ perspective. Raising awareness among HPs, patients, and partners may better support couples in managing sexual dysfunction and mitigate the negative impact.

Despite significant advancements in pharmacological treatment of IA [32,33], partners in our study experienced a negative emotional impact of living with a person with IA, like reported in previous studies [13,24,25]. Partners described their biggest challenge was to witness pain, fatigue, and limitations imposed by arthritis on their loved one, and simultaneously feeling the accompanying sense of powerlessness in their own role. The partners’ experience of powerlessness and why it may be so challenging can be understood through psychological mechanisms of perceived loss of control. In line with Bandura’s [34] concept of Perceived Controllability, the belief that one can influence events that affect one´s life, our findings suggest that the partners in this study experience a low sense of control. From their descriptions, it appears that they perceive little trust in their ability to modify the environment or influence the suffering of the person living with IA. This perceived lack of control may hinder them from taking constructive actions and can contribute to feelings of stress, frustration, and sadness. Thus, indicating a need for HPs to support partners in gaining a higher degree of self-efficacy in their own role as a partner. However, it may also be understood from an existential perspective. According to Frankl’s Logotherapy, the partners’ experience of sadness and helplessness when watching a loved one suffer is not meaningless. It is the price of love and proof that the partners are deeply connected to the person diagnosed with IA, and thus not a sign of failure or lack of control [35]. Supporting these partners should according to Frankl focus on helping partners recognise this, as it may help the partners transform helplessness into purpose and resilience, by choosing how they want to relate to the IA.

The way partners in the study chose to react and sought meaning was primarily through delivering practical support to the person with IA. All partners participating in the study described caring (emotionally committed to the well-being of the person with IA); however, the degree of caregiving (the actions partners took to protect or enhance the person with IA’s well-being) varied. According to Pearlin et al [36], caregiving should be understood as a natural part of any relationship and should not be considered a specific ‘role’ partners undertake. However, in the case of long-term illness and disability, Pearlin et al [36] proposes that this balance may change, and thus the person with IA becomes increasingly dependent, and the partners may take on more caregiving responsibilities, which over time can overtake the relationship and cause major emotional and physical stress. This aligns with our partners’ different descriptions of providing support; they described it from a natural part of being in a relationship, to some describing themselves as nurses, where the caregiving element was a larger part of their relationship, and the person with IA was more dependent on their help. In a Danish national cross-sectional questionnaire study from 2024 among 26,274 adults, 45 % were identified as an informal caregiver [37]. The caregivers reported powerlessness as the most burdensome factor, which was associated with elevated stress levels and an increased risk of both physical and emotional burden. These findings align with our study, in which some partners described their situation as intermittently stressful. Further, in a meta-analysis based on observational studies from 2003 [38], caregiving of elders has been associated with elevated levels of both stress and depression, lower levels of well-being and physical health [38]. Based on our findings, we cannot conclude on the physical and emotional health implications of being a partner to a person with IA; however, our findings imply grounds for further research on the negative impact of caregiving and possible preventive measures.

In our study, it varied how partners managed the different challenges, although knowledge was described as overall important. Acceptance, collaboration, maintaining a positive mindset, and balancing the different needs within the family were described as ways to manage. However, this did not come naturally; partners reported that it was a skill they had to acquire. This highlights the importance for both the person diagnosed with IA and their partners to have access to relevant knowledge and support, enabling them to develop self-management skills [9] and find meaning [35].

To discuss the strength and limitations of this study, we will apply the 4 principles of trustworthiness as described by Lincoln et al [39]. We believe we have demonstrated credibility by collecting data both from FG and individual interviews, allowing for different experiences and perspectives to resurface and be discussed. Developing the interview guide in collaboration with research partner couples, involving multiple researchers in coding and theme developing, and presenting participant quotations helped ensure that interpretations were grounded in participants’ experiences and thoroughly explored during the FG and interviews.

Looking back, some methodological changes could have improved the study’s credibility. First, the interview guide did not ask about the experience of the relationship and life before arthritis, which potentially could have provided valuable insight. Second, the study investigated a broad group of partners. Our findings indicate that perhaps it could have been relevant to investigate partners’ experience closer to the time of diagnosis, and to separate the three diagnostic groups. Lastly, while some may question the use of mixed interview modes, Johannessen et al. [40] note that such flexibility can enhance openness, consistent with our experience in this study. Although our constructivist approach acknowledges the role of researcher subjectivity, the detailed study descriptions provide transparency for others to follow our analytical steps. Engaging in reflexive discussions during the analysis, we remained attentive to how our perspectives might shape the interpretation while keeping the findings grounded in the data. Given the diversity within our sample of partners, and the central challenge of witnessing a loved one’s pain while feeling powerless, we consider the findings particularly transferable to partners of people living with chronic pain in other rheumatology departments. However, a limitation of this study is that the recruitment of partners relied on the person with IAs willingness to allow the researcher to contact their partner. Of 19 patients, only 12 provided such consent, often due to personal relationship reasons within the couple, resulting in a smaller and potentially more selective sample of partners. Thus, those who agreed to have their partner participate may represent couples who have managed IA more successfully, whereas those experiencing greater relational challenges may be underrepresented. Consequently, the findings may reflect the experiences of couples who are relatively well-functioning in their adjustment to IA. Thus, the transferability of these insights may be limited to contexts where similar resources in managing IA are available.

## CONCLUSION

Our findings suggest that partners experience a range of negative emotions and powerlessness related to witnessing their loved one´s suffering. Partners experience their own role as being supportive, with providing a varied degree of caregiving. Learning to manage life as a partner does not come easily. It is a skill, described as a form of ‘mental gymnastics’ requiring knowledge, skills training, and perhaps support to manage these challenges. These findings highlight the importance of providing partners with education and resources to help them self-manage life and their role as a partner to a person with IA.

## CRediT authorship contribution statement

**Charlotte Werdal Hansen:** Conceptualization, Data curation, Formal analysis, Funding acquisition, Investigation, Methodology, Visualization, Writing – original draft. **Kadija El Awsy:** Formal analysis, Writing – review & editing. **Michelle Josepsson Scott:** Formal analysis, Writing – review & editing. **Julie Midtgaard:** Conceptualization, Methodology, Supervision, Validation, Writing – review & editing. **Annette de Thurah:** Conceptualization, Methodology, Supervision, Validation, Writing – review & editing. **Pernille Fevejle Cromhout:** Conceptualization, Funding acquisition, Methodology, Supervision, Validation, Writing – review & editing. **Bente Appel Esbensen:** Conceptualization, Formal analysis, Funding acquisition, Investigation, Methodology, Supervision, Validation, Writing – review & editing.

## Competing interests

CWH reports financial support was provided by Danish Rheumatism Association, whom did not have any influence on the study object, methodology or analysis. All other authors declare they have no competing interests.

## Appendix Supplementary materials

Image, application 1

## Footnotes

Supplementary material associated with this article can be found, in the online version, at doi:10.1016/j.ero.2026.03.008.
