# Supplementary material for: Partners’ experiences of inflammatory arthritis impact on their lives: a qualitative study
Source: EULAR Rheumatol Open. 2026 Apr 2;2(3):100160. doi: 10.1016/j.ero.2026.03.008 (PMC13650337; doi:10.1016/j.ero.2026.03.008)
Supplement: Supplementary file 1 [file mmc1.pdf]

Supplementary file:
Interview guide for the partners

Icebreaker
What is the best holiday, you've been on?

Introduction
- Presentation of the participants
- Presentation of researcher and observer
- Purpose of interview /FG
- FG: We will present you with different statements that do not necessarily express our opinion, but will form the basis for our discussion
- No wrong answer
- The framework of the FG/interview (max 2 hours)
- Phones on silent
- Confidentiality, anonymity and data security, how to withdraw.
- Recorded electronically
- Demographic Questionnaire

Theme	Statement	Open-ended questions
Partner's role	1. We deal with my partner's arthritis, treatment and symptoms together<br>2. My partner's arthritis has nothing to do with me	• What is your role as a partner to a person with arthritis?<br>• How can you best help your partner?
Communication and relationships	1. We talk every day about the arthritis and how my partner is doing today<br>2. I often prioritize my partner's needs over my own<br>3. I have been considering divorce/breaking up due to my partner's illness	• What is the biggest challenge of being a partner to someone with arthritis?<br>• How much do you worry about your partner?<br>• How does arthritis affect your communication?<br>• How does arthritis affect your relationship?<br>• How does arthritis affect your children?
Knowledge	1. As a partner of someone with arthritis, you need knowledge about the illness and treatment to the same degree as the person who has arthritis<br>2. Questions about my partner's arthritis is best answered through my partner	• What knowledge do you need when you are a partner?<br>• How would you like to get the knowledge you need?
Support and involvement	1. As a partner, I should not be involved in consultations at the Hospital<br>2. I contribute with important knowledge about how my partner is doing and should participate<br>3. I know where I can ask for help in case, I need it	• What would you like healthcare professionals to be able to help you with as a partner?<br>• What are your thought about participating in consultations at the Hospital?<br>• What are your thoughts about being invited to a partner event? Or couple event?<br>• How should/can healthcare professionals support you?

Ending
- Do you have any questions? Anything you would like to add/elaborate on that I did not ask about?
- Thank you for talking and for coming
